# Clinical profile in *KMT2A-SEPT6-*positive acute myeloid leukemia: Does it often co-occur with *NRAS* mutations?

**DOI:** 10.3389/fmed.2022.890959

**Published:** 2022-09-21

**Authors:** Fang Chen, Ying Yang, Shuang Fu

**Affiliations:** ^1^Department of Hematology Laboratory, Shengjing Hospital of China Medical University, Shenyang, China; ^2^Division of Hematology, Department of Medicine, Shengjing Hospital of China Medical University, Shenyang, China

**Keywords:** acute myeloid leukemia, gene rearrangement, *KMT2A-SEPT6*, mutations, *NRAS*

## Abstract

**Background:**

The *KMT2A-SEPT6* fusion gene is a relatively rare genetic event in leukemia. Its clinical characteristics and prognosis, especially the profile of co-occurring gene mutations remain unclear.

**Methods:**

We retrospectively analyzed the characteristics of four cases carrying *KMT2A-SEPT6* in our hospital, and provided a literature review.

**Results:**

All the four patients were diagnosed with acute myeloid leukemia (AML) and harbored X chromosome and 11 chromosome rearrangements, they all manifested high levels of D-dimer. Three of four patients had *NRAS* mutations while one patient with congenital AML did not. Of the four cases, one developed drug resistance, one suffered relapse after bone marrow transplantation (BMT) and two died. Combined with other cases reported in the literature, we found that of all patients diagnosed with AML, 90.9% were children (≤9 years old). Patients with white blood cells ≥20.0 × 10^9^/L or diagnosed with M4 had a shorter overall survival (*P* < 0.05). Age, whether to receive BMT, and the chromosome rearrangement patterns had no significant effect on overall survival (*P* > 0.05).

**Conclusions:**

*KMT2A-SEPT6* was more commonly observed in pediatric AML patients, some of which may co-occur with *NRAS* mutations. The prognosis was related to the white blood cell levels and the leukemia subtype, but was not related to age or BMT. More cases need to be accumulated to better understand the profile in *KMT2A-SEPT6*-positive AML.

## Introduction

Acute myeloid leukemia (AML) is a malignant tumor that originates from myeloid blood cells and is characterized by abnormally increased leukemic cells in bone marrow or peripheral blood (1). Cytogenetic and molecular abnormalities commonly occur in AML patients. Based on genetic mutations and specific chromosomal rearrangements, the World Health Organization (WHO) divides AML with recurrent genetic abnormalities into different subgroups (2). Translocations involving the lysine (K)-specific methyltransferase 2A gene (*KMT2A*), previously known as mixed lineage leukemia (*MLL*), are common chromosomal abnormalities in AML (3, 4). The *KMT2A* gene at 11q23 has many partner genes, of which over 80 have been identified (5).

Among them, *SEPT6* located at Xq24 which is involved in the formation of the *KMT2A* arrangement t(X;11)(q22-24;q23), is extremely rare in AML (6). *SEPT6* is a member of Septins family, an evolutionarily conserved family of GTP-binding proteins that associate with cell polarity, cytokinesis and oncogenesis (7, 8). Five septin genes (*SEPT2, SEPT5, SEPT6, SEPT9*, and *SEPT11*) were identified as *KMT2A* fusion partner (9). *SEPT6*, which plays a role in actin and microtubule cytoskeletons, is involved in infectious diseases, Down's syndrome, schizophrenia, bipolar disorder and cancers, including leukemia and lymphoma (10, 11). To our knowledge, only a limited number of *KMT2A-SEPT6*-positive cases have been documented in the literature. Most cases are children while only one adult case has been reported (6, 12–21). The exact role of *KMT2A-SEPT6* in hematopoietic cells and its effect on leukemogenesis are still unknown. There is little information on the clinical features, treatment strategies and prognosis of such patients, and accompanying gene mutations carried by such patients have not been described.

We retrospectively analyzed data from four acute leukemia patients carrying the *KMT2A-SEPT6* fusion gene who were treated in our hospital, especially gene mutation information. Additionally, we reviewed cases in the literature together with our cases to provide evidence for potential therapeutic strategies.

## Materials and methods

### Case selection

We collected four patients harboring the *KMT2A-SEPT6* gene from a pool of 1,656 leukemia patients within our hematological disease database in the past 4 years, which were referred to as cases 1–4. The diagnostic criteria were according to the WHO classification of tumors of hematopoietic and lymphoid tissues (22). We conducted a retrospective analysis and systematic summary with information on morphology, flow cytometric analysis, cytogenetics, molecular biology, and other related laboratory test results.

### Literature review

We conducted a literature search on PubMed with the keywords “*KMT2A-SEPT6*,” “*MLL-SEPT6*,” “*KMT2A-SEPTIN6*,” “*MLL-SEPTIN6*” or “t(X;11)” to gather related case reports.

### Statistical analysis

Kaplan–Meier method was used for survival evaluation. The log-rank test was used to assess the difference between groups. *P* < 0.05 was considered statistically significant. All data were analyzed with SPSS Statistics, version 21 (StatSoft).

## Results

### Clinical presentation

All *KMT2A-SEPT6*-positive cases (cases 1–4) were male with ages ranging from 0 to 57 years, and their detailed information is summarized in Table 1. All cases had manifestations of fever and pale complexion. The three pediatric cases (cases 2, 3, and 4) were accompanied by hepatosplenomegaly, and two cases (cases 3 and 4) had scattered petechiae and ecchymoses. Case 1 was an elderly male patient with perianal abscess and diabetes in addition to the above symptoms. Case 2 was accompanied by pain in both lower limbs, tenderness of the sternum and hypertrophy of the tonsils. In case 3, multiple lymph nodes were palpable on the bilateral neck, he was positive for sternal tenderness, and he also had hyperuricemia and acute bronchial pneumonia. Case 4 was a newborn delivered by cesarean section due to a “decreased fetal heart rate.” The birth weight was 3,100 g. The infant had no spontaneous breath at birth and was generally cyanotic. He restored spontaneous respiration under assisted ventilation, and he developed persistent pulmonary arterial hypertension and neonatal pneumonia. His parents were healthy and had no history of genetic disease.

**Table 1:** Clinical features of four *KMT2A-SEPT6*-positive AML patients.

**Items**	**Case 1**	**Case 2**	**Case 3**	**Case 4**
Sex	Male	Male	Male	Male
Age	57 years	9 years	16 months	0 month
Physical examination	Perianal abscess	Lower limbs pain, sternal tenderness, and tonsil hypertrophy	Scattered petechiae on the neck, cervical lymphadenopathy and sternal tenderness (+)	Scattered petechiae and ecchymoses
Hepatomegaly/splenomegaly	No/No	Yes/Yes	Yes/Yes	Yes/Yes
CNS involvement	No	Yes	No	No
WBC/Hb/PLT (× 10^9^/L/g/L/ × 10^9^/L)	12.3/79.0/105.0	3.0/93.0/245.0	123.8/41.0/39.0	112.0/90.0/9.0
Serum LDH (U/L)	NA	966	2,080	NA
D-Dimer [μg/L(DDU)]	13117.0	3149.0	2469.0	620.0
Blood blasts (%)	69.0	2.0	52.0	17.0
Bone marrow blasts (%)	92.0	27.5	56.0	20.4
Morphological diagnosis	AML-M5	AML-M2	AML-M4	AML-M5
Immunophenotype	The leukemic cells expressed HLA-DR, CD117, CD33, CD13, CD38, CD15, CD64, and CD4	The leukemic cells expressed HLA-DR, CD33, CD38, CD15, CD64, and CD4	The leukemic cells expressed HLA-DR, CD33, CD13, CD38, CD15, and CD64	The leukemic cells expressed HLA-DR, CD33, CD38, CD15, and CD64
Karyotype	46, Y, t(X;11)(q24;q23)[4]/46,XY[6]	45,Y,del(X)(q21),der(11) t(X;11)(q24;q23),-20,add(22)(q13)[3]	46, Y, t(X;11)(q24;q23), del(7)(q21q31)[13]/46,XY[2]	46,Y,ins(X;11) (q23;q24q12)[10]
Gene mutations	NRAS: NM_002524:exon2:c.G35T: p.G12V, VAF = 0.04, Missense; ASXL1: NM_015338:exon12:c.2464 dupA:p.D821fs, VAF = 0.36, Frameshift	NRAS: NM_002524:exon2:c. G35T:p.G12V, VAF = 0.03, Missense	NRAS:NM_002524:exon4:c. G436A:p.A146T, VAF = 0.44, Missense	Negative
Treatment protocol	IA regimen (resistance); then changed to decitabine combined with half-dose CAG regimen (once) + high dose cytarabine	MAE regimen + intrathecal chemotherapy + BMT	HA regimen	No chemotherapy
CR	Yes	Yes	Yes	NA
Relapse	Yes	Yes	Yes	NA
Follow-up (months)	18	43	36	0.5
Outcome	Alive	Alive	Died	Died

CNS, central nervous system; WBC, white blood cells; Hb, hemoglobin; PLT, platelets; LDH, lactate dehydrogenase; VAF, variant allele frequencies; CAG, cytarabine + aclacinomycin + granulocyte colony stimulating factor; MAE, Mitoxantrone + cytarabine + etoposide; BMT, bone marrow transplantation; HA, homoharringtonine + cytarabine; CR, complete remission; NA, not available.

Laboratory results showed that three cases (cases 1, 3, and 4) had high white blood cell (WBC) counts, anemia, and low platelet counts. One patient (case 2) had low hemoglobin levels and WBC counts but normal platelet counts, and two patients (cases 2 and 3) had increased serum lactate dehydrogenase levels. The D-dimer levels of all four patients were increased.

### Morphological evaluation

**Figure 1:**
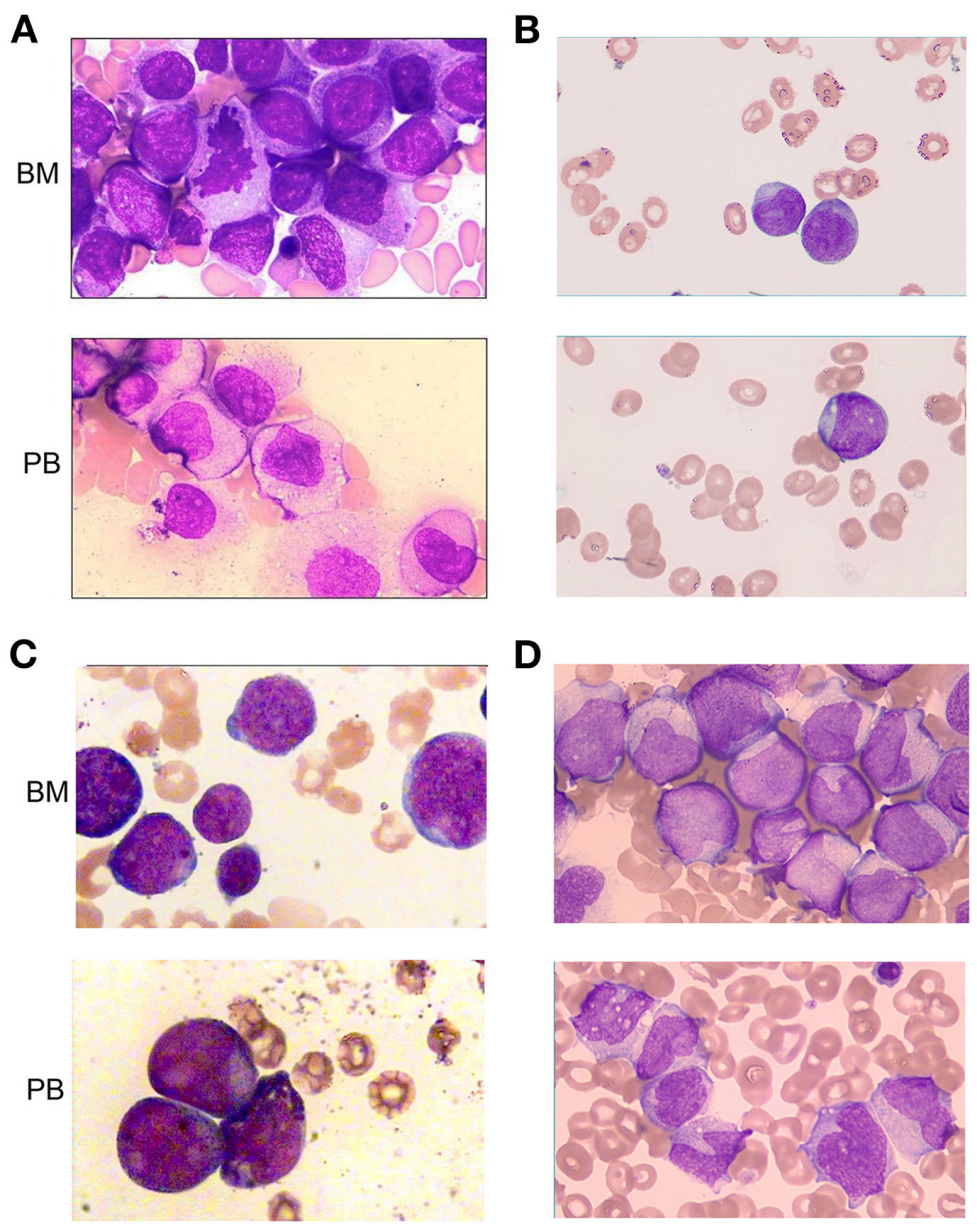
Morphologic evaluation of leukemic cells at diagnosis (Wright–Giemsa stain, × 1,000). **(A–D)** represent cases 1, 2, 3, and 4, respectively. BM, bone marrow; PB, peripheral blood.

All cases exhibited morphological characteristics of AML (Figure 1). Leukemia cells have the morphological characteristics of blast cells, including medium to large nuclei with round and often eccentric nuclei, loose chromatin, abundant basophilic cytoplasm, and Auer bodies can be seen in some cells. The French American British (FAB) morphological classification of each case was M5 (cases 1 and 4), M2 (case 2) or M4 (case 3). Three cases (cases 1, 3, and 4) showed hypercellular bone marrow, and the other case (case 2) had severe hypocellular bone marrow. Case 1 and case 4 were evaluated as M5 and revealed 92.0 and 20.4% blasts in marrow aspirate and 69.0 and 17.0% blasts in peripheral blood, respectively (Table 1; Figures 1A,D). Case 2 was evaluated as M2; the marrow aspirate revealed 27.5% myeloblasts and the peripheral blood exhibited 2.0% blasts (Table 1; Figure 1B). Case 3 was evaluated as M4; the marrow aspirate showed 56.0% blasts and the peripheral blood exhibited 52.0% blasts (Table 1; Figure 1C).

### Flow cytometric analysis

**Figure 2:**
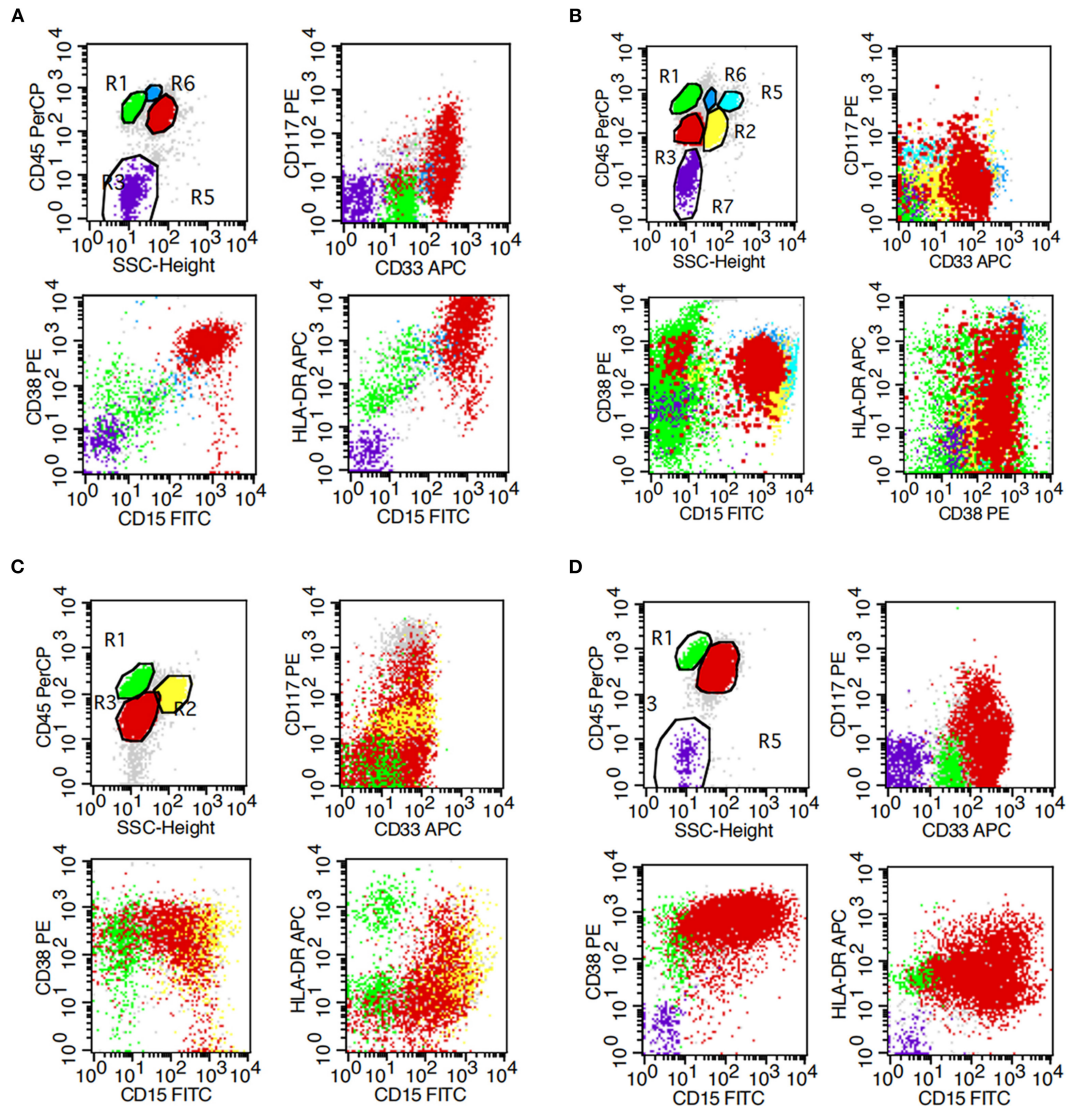
Flow cytometry results of bone marrow. **(A–D)** represent cases 1, 2, 3, and 4, respectively.

Flow cytometric analysis revealed the presence of myeloid blasts in bone marrow samples from all four patients (Table 1; Figure 2). The percentage of blasts was highest at 92% in case 1 and lowest at 4.2% in case 2. All cases were positive for CD33, CD15, and CD64, indicating myeloid lineage, and CD13 was positive only in case 1 and case 3. All the cases were positive for CD38 and HLA-DR. CD117 was positive only in case 1, and CD34 was negative in all four cases.

### Cytogenetic analysis

**Figure 3:**
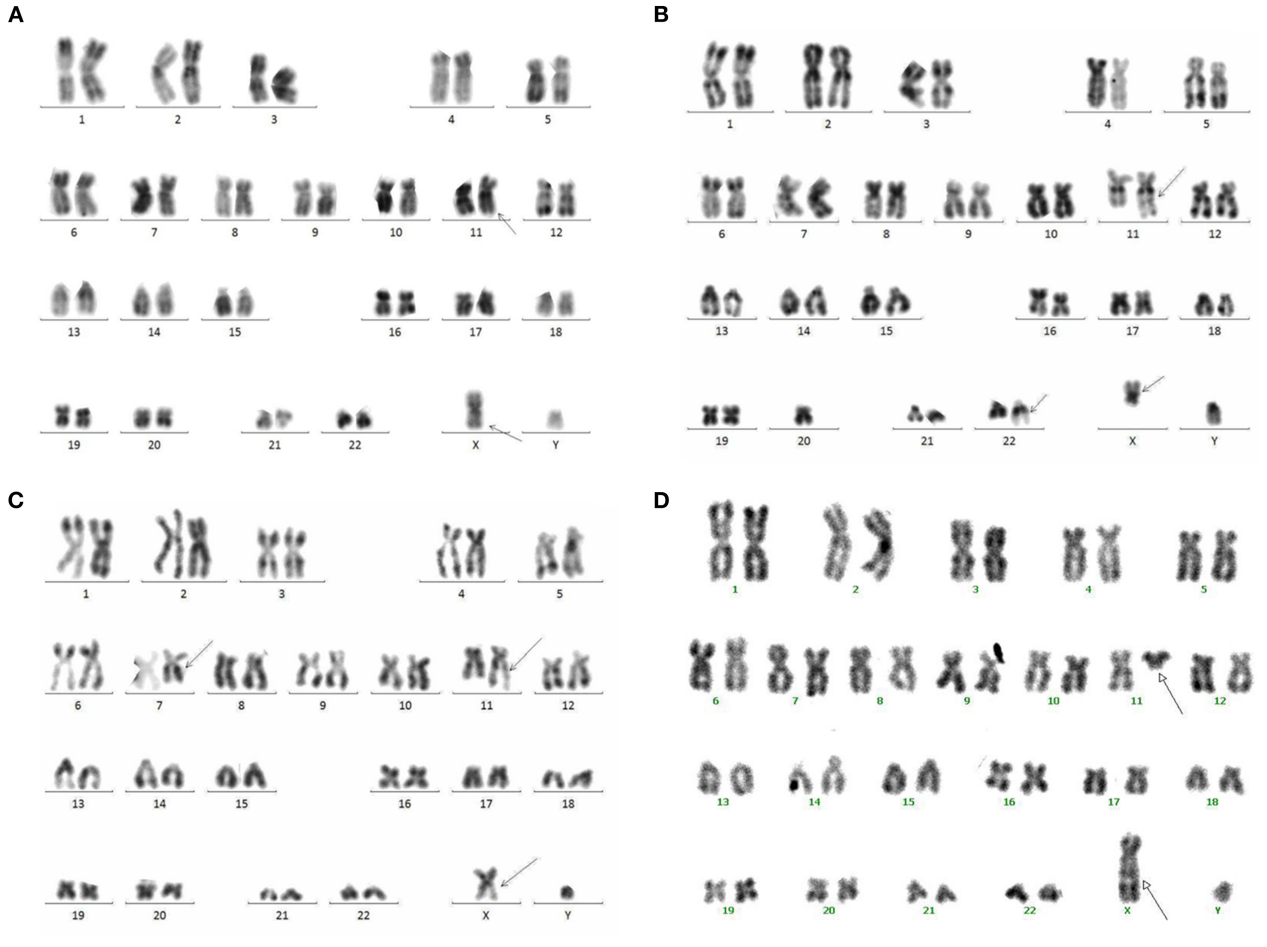
Karyotype analysis results of bone marrow. **(A–D)** represent cases 1, 2, 3, and 4, respectively.

The results showed that all four cases had clonal abnormalities of the X chromosome and chromosome 11 or complex karyotype abnormalities (Table 1; Figure 3). In case 1, the metaphase cells exhibited abnormalities of t(X;11)(q24;q23) (Figure 3A). The metaphase cells collected in case 2 showed 45, Y, del(X)(q21), der(11)t(X;11)(q24;q23), −20, add (22)(q13) (Figure 3B). In case 3, in addition to the abnormal karyotype of t(X;11)(q24;q23), thirteen metaphase cells had del(7)(q21q31) (Figure 3C). The metaphase cells in case 4 showed an abnormal karyotype of 46, Y, ins(X;11)(q23;q24q12) (Figure 3D).

### Molecular analysis

We performed molecular biology tests, including screening of fusion genes and next-generation sequencing (NGS) analysis, on all cases (Table 1). The *KMT2A-SEPT6* fusion gene was detected in all four cases. The NGS panel included a total of 20 frequently mutated genes in AML: *ASXL1, CEBPA, DNMT3A, EZH2, FLT3, IDH1, IDH2, KIT, NPM1, PHF6, RUNX1, TET2, TP53, BCOR, GATA2, KMT2A, KRAS, NRAS, PDGFRA*, and *WT1*. The sequencing depth was 2,000 ×. The results showed that three cases (cases 1–3) harbored *NRAS* (NM_002524) mutations, and the mutation sites of cases 1 and 2 were both *NRAS* G12V, with variant allele frequencies (VAFs) of 0.04 and 0.03, respectively. In addition, case 1 was also accompanied by an insertion mutation of ASXL1 D821 with a VAF of 0.36. The reexamination results at the third month showed that the *KMT2A-SEPT6* gene remained positive but its expression level dropped from 100 to 24.4%. The *ASXL1* D821 VAF dropped to 0.01, and the *NRAS* G12V gene mutation was not detected. Four months later, the patient achieved complete remission (CR), the *ASXL1* D821 VAF dropped to 0.003, and both the *NRAS* mutation and *KMT2A-SEPT6* fusion gene were negative. The *NRAS* A146T mutation in case 3 occurred in exon 4 with a VAF of 0.44. *KMT2A-SEPT6* gene and *NRAS* gene mutations both turned negative on the ninth month after diagnosis. The patient relapsed on the twenty-seventh month with positive *KMT2A-SEPT6* and *NRAS* A146T mutation (VAF = 0.27). In case 4, no pathogenic gene mutations were detected.

### Clinical course

The treatment and follow-up information of all cases (cases 1–4) is shown in Table 1. Three patients (cases 1–3) received chemotherapy, patient 2 subsequently received a bone marrow transplantation (BMT), and patient 4 was a newborn and did not receive any chemotherapy. The clinical follow-up period ranged from 0.5 to 43 months, with a median of 27 months. Case 1 received induction chemotherapy of IA (idarubicin + standard cytarabine) regimen, and revealed non-remission with blasts from 92.0 to 40.5% in bone marrow. Then, he received D-CAG (decitabine combined with low-dose cytarabine + aclacinomycin + granulocyte colony stimulating factor) and achieved CR. The *KMT2A-SEPT6* gene expression reduced from 100 to 24.4%, and the *NRAS* G12V gene mutation was negative after the second chemotherapy. Subsequently, he received high-dose cytarabine consolidation therapy and an intrathecal injection (cytarabine + dexamethasone + methotrexate) for central nervous system infiltration prevention. The patient revealed a normal karyotype and negative molecular results of *KMT2A-SEPT6* rearrangement and *NRAS* mutation, and sustained CR for 10 months. However, he relapsed 3 months later. From this time point the patients had a stable blast count of 5.0%-10.5% to the last follow-up. Case 2 received MAE (mitoxantrone + standard cytarabine + etoposide) regimen and reached CR. Then he received high-dose cytarabine consolidation therapy. After that, the patient received allogeneic hematopoietic stem cell transplantation (allo-HSCT). The conditioning regime was modified BU/CY + ATG (busulfan/cyclophosphamide + antithymocyte globulin, peking regime). The donor was his father, HLA 5/10, A+ to B+. We used CsA + MTX + MMF (cyclosporine + methotrexate + mycophenolate mofetil) for Graft-Versus-Host Disease (GVHD) prophylactic. The process of transplantation was well-off, and bone marrow assessment results were negative continuously. However, 19 months after transplantation, he relapsed with central nervous system leukemia. Following five courses of intrathecal injection, the child achieved CR again. Case 3 received HA (homoharringtonine + standard cytarabine) regimen and intrathecal injection (cytarabine + dexamethasone + methotrexate). He reached CR 4 weeks after diagnosis. This patient relapsed 27 months later and died 36 months after diagnosis. Case 4 had dyspnea at birth, and he was on assisted ventilation and given blood infusion to improve anemia and thrombocytopenia. The newborn's condition did not improve during the treatment, and the child died 2 weeks later.

### Literature review

A total of 22 *KMT2A-SEPT6*-positive cases were included in this literature review, including four cases in our report and eighteen cases from the literature (6, 12–21). Tables 2, 3 list the detailed clinical information of all patients and clinical features of evaluable patients.

**Table 2:** Detailed characteristics of *KMT2A-SEPT6* positive AML cases reported in the literature.

**Patient No**.	**Sex**	**Age**	**Hepatomegaly** **/splenomegaly**	**CNS involvement**	**WBC/Hb/PLT (× 10^9^/** **L/g/L/ × 10^9^** **/L)**	**Blood blasts** **(%)**	**Bone marrow blasts (%)**	**Immunophenotype**	**Karyotype**	**Diagnosis**	**Treatment protocol**	**Treatment outcome**	**Survival months**	**Reference**
1	M	57 years	No/No	No	12.3/79.0/105.0	69.0	92.0	The leukemic cells expressed HLA-DR, CD117, CD33, CD13, CD38, CD15, CD64, and CD4.	46, Y, t(X;11)(q24;q23) [4]/46,XY[6]	AML-M5	IA regimen (resistance); then changed to decitabine combined with half-dose CAG regimen + high dose cytarabine	Resistance 2 months after diagnosis, CR 10 months after changing chemotherapy	18	Present
2	M	9 years	Yes/Yes	Yes	3.0/93.0/245.0	2.0	27.5	The leukemic cells expressed HLA-DR, CD33, CD38, CD15, CD64, and CD4.	45,Y,del(X) (q21),der[11] t(X;11)(q24;q23),-20,add[22](q13)[3]	AML-M2	MAE regimen + intrathecal chemotherapy + BMT	CR 6 months after diagnosis	43	Present
3	M	16 months	Yes/Yes	No	123.8/41.0/39.0	52.0	56.0	The leukemic cells expressed HLA-DR, CD33, CD13, CD38, CD15, and CD64.	46, Y, t(X;11)(q24;q23), del[7] (q21q31)[13] /46,XY[2]	AML-M4	HA regimen	Died 34 months after diagnosis	36	Present
4	M	0 month	Yes/Yes	No	112.0/90.0/9.0	17.0	20.4	The leukemic cells expressed HLA-DR, CD33, CD38, CD15, and CD64.	46,Y,ins(X;11) (q23;q24q12)[10]	AML-M5	No chemotherapy	Died 0.5 month without chemotherapy	0.5	Present
5	F	3 months	NA	NA	163.2/NA/NA	NA	NA	The leukemic cells expressed CD4, CD13, CD15, and CD33.	46, XX, t(5,11) (q13;q23), add(X)(q22) [12]/46, XX, t(5,11)(q13;q23) [6]/47, XX, t(5,11), add(X)(q22), +add(X) [1]/46, XX [1].	AML-M2	Chemotherapy + BMT	Died 9 months after diagnosis	9	(6)
6	M	7 months	NA	NA	608.0/NA/NA	NA	NA	The leukemic cells expressed CD4, CD13, and CD33	46, XY, ins(X;11)(q22-24;q23)	AML-M2	Chemotherapy + BMT	CR 35 months after diagnosis	35	(6)
7	F	6 months	NA	NA	58.5/NA/NA	NA	NA	The leukemic cells expressed CD4, CD13, CD33, and HLA-DR.	46, X, add(X)(q2?), del(11q?)[20]	AML-M1	Chemotherapy + BMT	Died 11 months after diagnosis	11	(6)
8	F	6 months	NA	NA	280.0/NA/NA	NA	NA	NA	46,XX,ins(X;11) (q24;q23)	AML-M2	AML-BFM 98 protocol	CR 13 months after diagnosis	13	(12)
9	F	20 months	Yes/Yes	Yes	397.0/anemia/ thrombocytopenia	NA	91.0	The leukemic cells expressed CD33, CD15, CD11b, and HLA-DR.	47,X, der(X) t(X; 11) (q22; q23) t(3,11) (p21; q12), der[3] t(3,11) (p21;q23)t(X;11) (q22;q25), + 6, der(11)del(11) (q12?qter)	AML-M4	Chemotherapy	Died during induction chemotherapy	NA	(13)
10	M	10 months	NA	NA	13.4/anemia/ thrombocytopenia	13.0	NA	The leukemic cells expressed CD11, CD13, CD15, CD33, and did not express lymphoid antigens.	46,Y,t(X;11) (q22;q23) [25]/46,XY[5]	AML-M2	CCG 2891 protocol + haploidentical transplant	PR 4 months after treatment, CR 7 years after diagnosis	84	(13)
11	M	29 months	NA	NA	NA	NA	74.2	The leukemic cells expressed CD13, CD33, HLA-DR, TdT, and CD7	46,Y,inv ins(X;11)(q24;q23q13)[13]/46,XY[7]	AML-M5	BHAC regimen	CR 1 month after diagnosis	8	(14)
12	M	8 months	Yes/Yes	Yes	112.0/84.0/41.0	63.5	90.0	The leukemic cells expressed CD33, CD15, CD11b and CD36, and negative for CD34, CD13, CD14, CD7, CD19, and TdT.	46,Y,ins(11; X)(q23; q24q24)	AML-M4	1-β-D-arabinofurano sylcytosine + daunomycin + etoposide + intrathecal chemotherapy	CR 1 month after diagnosis, died 5 months after relapse	8	(15)
13	M	3 years	NA	NA	NA	NA	NA	NA	46,Y,t(X;11) (q22;q23)	AML M2	BMT	CR 97 months after diagnosis	96.9	(16)
14	M	12 months	NA	NA	NA	NA	NA	NA	46,Y,t(X;11) (q13;q23)	AML M4	BMT	Died 24.4 months after diagnosis	24.4	(16)
15	M	12 months	NA	NA	NA	NA	NA	NA	46,Y,t(X;11) (q24;q23)	AML M5	Chemotherapy	CR 101.2 months after diagnosis	101.2	(16)
16	M	6 years	NA	NA	At diagnosis: 1.6/84.0/ 254.0 At relapse: 9.5/ 115.0/85.0	At diagnosis: NA At relapse: 40.0%	At diagnosis: NA At relapse: 88.0%	The leukemic cells expressed CD4 and CD13, and did not express HLA-DR, CD33, CD11b and other lymphocyte-associated antigens; the cells at relapse expressed CD4, CD13 and CD33, and did not express HLA-DR, CD34, CD11b, and CD56	46,XY,t(X;11) (q24;q23)	AML	At diagnosis: doxorubicin, cytarabine, methotrexate, vincristine, and 6-mercaptopurine. At relapse, all-trans retinoic acid, G-CSF, cytarabine, etoposide, and Mitoxantron.	CR 61 months after diagnosis, died 11 months after relapse	72	(17)
17	F	12 months	No/No	No	16.4/93.0/81.0	28	82.0	NA	47,X,add(X) (p11),+6, add[11](q23)[20].ish der(X)add(X)(p11) ins(X;11) (q?;q23q23), der(11)ins(11;?) (q22;?)ins(X;11)	AML M2	ELAM 02 + BMT	CR 58 months after diagnosis	59	(18)
18	M	43 years	No/No	No	1.0/109.0/95.0	1	85.0	The leukemic cells expressed CD33 and MPO.	ins(X; 11)(q24-25; q23), del(11)(q23)	AML M5	Palliative care only	CR 25 months after diagnosis	31	(19)
19	F	17 months	NA	NA	NA	NA	NA	NA	47,X,add(X)(p11), +6,add(11) (q23)[20]	AML M2	NA	NA	NA	(20)
20	M	12 months	NA	NA	NA	NA	NA	NA	46,Y,t(X;11) (q24;q23) [11]/46,XY[9]	AML	NA	NA	NA	(20)
21	M	0 month	NA	NA	NA	NA	NA	NA	46,Y,ins(X;11) (q24;q13q23) [11]	AML	NA	NA	NA	(20)
22	M	13 months	NA	No	35.0/64.0/35.0	6.0	73.0	The leukemic cells expressed CD45, CD33, CD15, CD34, CD64, HLA-DR, and MPO. In addition, a small population of blasts with monocytic differentiation (CD 641 and CD141) was detected.	46,Y,ins (11;X)(q23; q24q22) [14]/46,idem, i[10](q10)[6]	AML M4	CCG 2891 protocol, Regimen C + intensive intrathecal chemotherapy + radiation to the extramedullary sites of disease + BMT	CR 2 months after diagnosis	6	(21)

M, male; F, female; CNS, central nervous system; WBC, white blood cells; Hb, hemoglobin; PLT, platelets; CAG, cytarabine + aclacinomycin + granulocyte colony stimulating factor (G-CSF); MAE, Mitoxantrone + cytarabine + etoposide; BMT, bone marrow transplantation; HA, homoharringtonine + cytarabine; BHAC, idarubicin + 1-β-D-arabinofuranosylcytosine + thioguanine; CCG, the Children's Cancer Group; AML-BFM, acute myeloid leukemia-Berlin-Frankfurt-Munster; CR, complete remission; PR, partial remission; NA, not available.

**Table 3:** Clinical features of evaluable patients.

**Characteristic**	***N*** **(%)**
**Gender**	***N*** **= 22**
Male	16 (72.7%)
Female	6 (27.3%)
**Age (years)**	***N*** **= 22**
≤1	12 (54.5%)
>1 and < 18	8 (36.4%)
≥18	2 (9.1%)
**WBC count (× 10** ^**9**^ **/L)**	***N*** **= 15**
≥20.0	9 (60.0%)
< 20.0	6 (40.0%)
**Symptom at presentation**	***N*** **= 10**
Hepatosplenomegaly	5 (50.0%)
CNS involvement	3 (30.0%)
Lymphadenopathy	3 (30.0%)
Skin involvement	2 (20.0%)
**FAB classification**	***N*** **= 22**
M1	1 (4.5%)
M2	8 (36.4%)
M4	5 (22.7%)
M5	5 (22.7%)
Unknown	3 (13.6%)
**Chromosomal abnormalities**	***N*** **= 22**
Translocations	11 (50.0%)
Insertions	9 (40.9%)
Complex abnormalities	3 (13.6%)
**Treatment protocol**	***N*** **= 19**
Chemotherapy alone	8 (42.1%)
BMT	9 (47.4%)
No chemotherapy	2 (10.5%)
**Survival outcome**	***N*** **= 19**
Alive	11 (57.9%)
Died	8 (42.1%)

WBC, white blood cells; CNS, central nervous system; BMT, bone marrow transplantation.

The age of the patients ranged from 0 to 57 years (median = 1 year), with a male–female ratio of nearly 3:1 (16 males vs. 6 females). Twenty patients (90.9%) were children (≤9 years old), including twelve (54.5%) infants (≤1 year old). The majority of the patients manifested leukocytosis (range 1–608 × 10^9^/L), anemia (range 41–109 g/L) and low platelet counts (range 9–254 × 10^9^/L). According to the high WBC index (23), nine (60.0%) of the fifteen cases with WBC count information were defined as high WBC levels. Twelve cases were not provided with a description of clinical features. Of the remaining 10 cases, five children (50.0%) had splenomegaly and hepatomegaly, and three patients (30.0%) had lymphadenopathy. Central nervous system involvement was observed in three children (30.0%), and skin involvement was observed in two cases (20.0%). All patients were diagnosed with AML (twenty children and two adults) according to the former FAB classification: five patients (three children and two adults, 22.7%) with M5, five children (22.7%) with M4, eight children (36.4%) with M2, one child (4.5%) with M1, and three children (13.6%) unknown. All the cases had available cytogenetic information, and chromosomal translocations (eleven cases) were the most common chromosomal rearrangements, followed by chromosomal insertions (nine cases). Among them, Xq24 (nine cases) and 11q23 (fourteen cases) were the most frequently involved chromosomal bands. Three cases (13.6%) demonstrated complex abnormalities. Eight patients (42.1%) received chemotherapy alone. Nine patients (47.4%) received BMT. Eight patients (42.1%) died.

**Figure 4:**
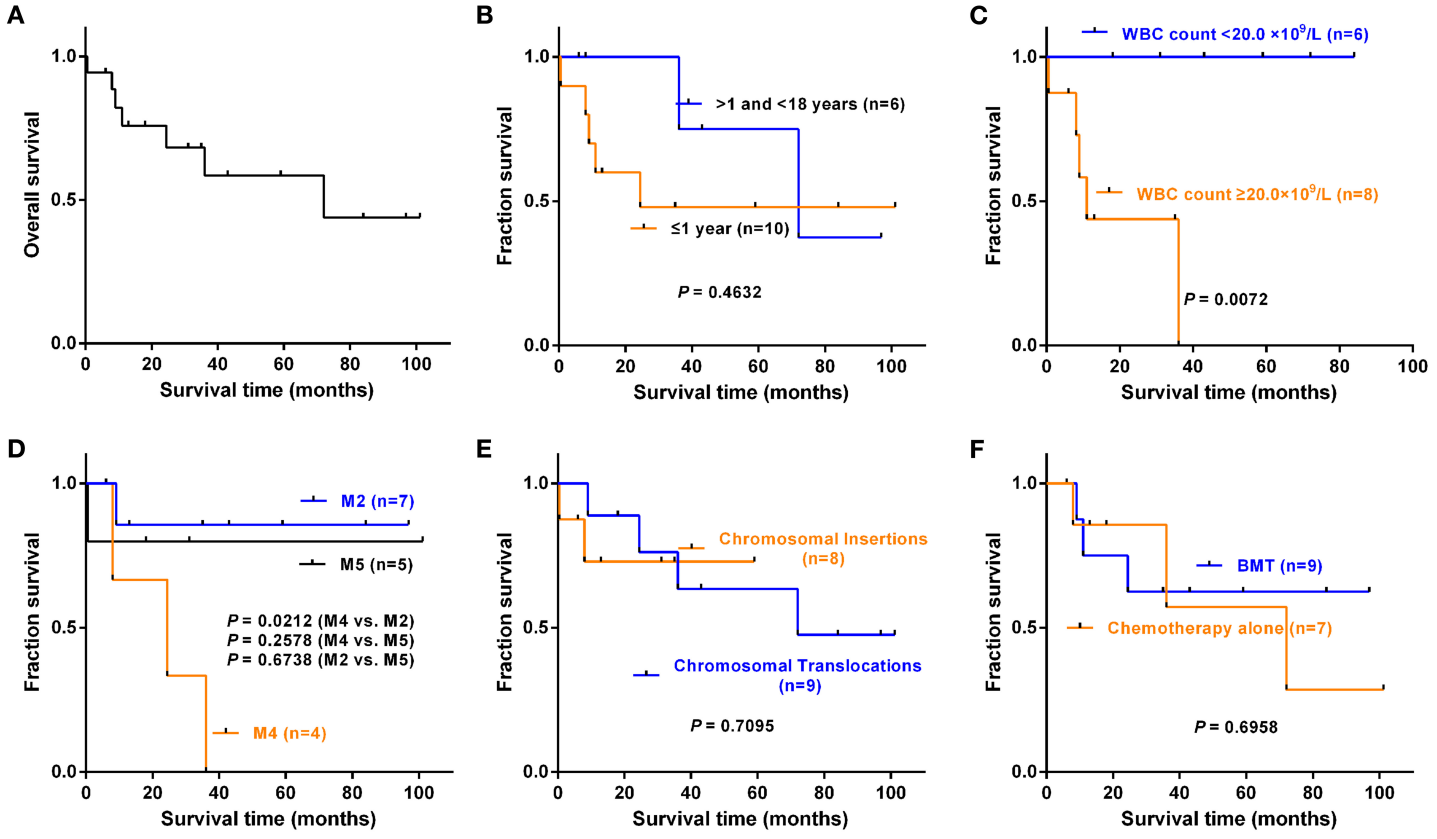
Kaplan–Meier survival analysis of eighteen cases with complete follow-up information. These included fourteen reported cases with clinical follow-up and four cases in our series. **(A)** Overall survival of eighteen cases. **(B)** Infant group (≤1 year) vs. pediatric group (>1 and < 18 years old), *P* = 0.4632. **(C)** WBC count < 20.0 × 10^9^/L vs. ≥20.0 × 10^9^/L, *P* = 0.0072. **(D)** FAB subtype. M4 vs. M2, *P* = 0.0212; M4 vs. M5, *P* = 0.2578; M2 vs. M5, *P* = 0.6738. **(E)** Chromosomal insertion vs. chromosomal translocation, *P* = 0.7095. **(F)** Chemotherapy alone vs. BMT, *P* = 0.6958.

Of all 22 cases, 18 cases had clinical follow-up with a median period of 27.7 months (0.5–101.5 months). Kaplan-Meier survival analysis was performed on eighteen cases with complete follow-up information (Figure 4A). As of the final follow-up, the median survival time was 72 months.

To understand the impact of different clinical features on overall survival (OS), we grouped the patients according to the characteristics in Table 3, and groups with fewer than three patients and cases with incomplete follow-up information were not included in the statistics. The results showed that there was no statistical significance in OS between the infant group (≤1 year old) and pediatric group (>1 and < 18 years old) (*P* = 0.4632, Figure 4B). The OS of patients with WBC levels ≥20.0 × 10^9^/L was much shorter than that of patients with WBC levels < 20.0 × 10^9^/L (*P* = 0.0072, Figure 4C). The patients with M4 had a shorter OS than those with M2 (*P* = 0.0212, Figure 4D). However, there were no significant differences in OS between the chromosomal translocation group and the chromosomal insertion group (*P* = 0.7095, Figure 4E) or between the patients who received chemotherapy alone and those who received BMT (*P* = 0.6958, Figure 4F).

## Discussion

The *KMT2A* gene is a frequent target of rearrangement in human leukemia, especially in infant and pediatric leukemia (14, 24, 25). These rearrangements include fusions with many partner genes but rarely involve the gene *SEPT6* on the X chromosome. The *KMT2A* gene and *SEPT6* gene are vulnerable to damage to form translocations associated with infant AML.

In this study, we described four cases of AML with the *KMT2A-SEPT6* fusion gene. The FAB subtypes were mainly M2, M4, and M5, which was consistent with the literature. To the best of our knowledge, only two adult patients have been reported, including one case in our series. Most of the cases that have been reported were children, and 54.5% were infant patients (≤1 year old). Among these cases, 60.0% had a high level of WBC, and 30% manifested central nervous system involvement, which were similar to the clinical features of *KMT2A*-rearranged AML patients. The findings of Balgobind et al. (26) showed that *KMT2A*-rearranged AML patients usually exhibit a high tumor burden, including organomegaly, a high median WBC count and central nervous system involvement. The present study included the largest number of *KMT2A-SEPT6* cases to date. The patients' NGS test results were not provided except ours, and we also tracked the patients' molecular biological examination results. Three of four cases in our series had *NRAS* mutations, while one case with congenital AML did not.

*NRAS* G12V is required in the leukemia self-renewal process, independent of its effects on growth and survival (27). Compared with other subtypes of leukemia, acute leukemia with *KMT2A* translocations (such as *KMT2A-AF4*, and *KMT2A-AF9*) harbored the fewest number of mutations, in which *NRAS* mutations commonly co-occur (27, 28). In our series, we identified *NRAS* mutations in *KMT2A-SEPT6-*positive AML patients for the first time, and most of the mutation sites appeared at codons 12 and 145. The former site is a hotspot mutant of *NRAS* and the latter site has also been reported (29, 30). The VAF of *NARS* mutation decreased as the patient's condition improved, indicating that there was no underlying residual clonal hematopoiesis after chemotherapy. When the patients achieved CR, they also turned negative. The underlying connection between *NRAS* mutations and *KMT2A-SEPT6* and whether non-congenital *KMT2A-SEPT6*-positive AML patients all have *NRAS* mutations remain to be further studied in a larger cohort in the future.

*KMT2A* gene rearrangement in AML usually indicates poor prognosis (5, 31). The prognostic significance of *NRAS* mutations in AML patients remains unclear (32–35). Of the four cases in our series, one developed drug resistance at first, one suffered relapse after BMT and two died, showing unsatisfactory therapeutic effect. However, whether the outcomes of patients with *KMT2A-SEPT6* were aggravated by the concurrence of *NRAS* mutations needs a follow-up study. Kaplan–Meier curves demonstrated that the pediatric group (>1 and < 18 years old) did not show better OS than the infant group (≤1 year old). Age may not be an independent prognostic factor for survival. Most of the patients received chemotherapy, nine of them received BMT, but three of them eventually died. The OS of patients between the chemotherapy alone group and the BMT group did not show a significant difference, which suggested that BMT may not improve the survival time of such patients. This was consistent with several studies and meta-analyses that suggested that BMT does not improve survival in patients with *KMT2A* rearrangement (36, 37). In addition, chromosome rearrangement patterns had no significant effect on the OS of patients. However, we found that a higher white blood cell count at the initial diagnosis was associated with a shorter OS. Moreover, FAB classification also has an impact on the prognosis of patients. A trend for worse OS was observed in M4 patients.

Our study has some limitations. First, this was a retrospective study, coupled with a limited number of reported cases and incomplete clinical information, resulting in small sample sizes in some subgroups, which may lead to false negative results. Second, our NGS detection only covers the twenty most frequently mutated genes in AML, and the prognostic effects of some critical genes may be neglected. Third, gene mutation information in the reported cases was not available and cases in our series were detailed but limited by sample size. Therefore, our findings need to be combined with more cases for further analysis in the future.

In conclusion, the *KMT2A-SEPT6* fusion gene was more commonly observed in pediatric patients diagnosed with AML. *NRAS* mutations were observed in these patients, most frequently of the *NRAS* G12V hotspot mutation. Whether *NRAS* mutations are related to the occurrence of *KMT2A-SEPT6*-positive AML is currently unclear. The prognosis was related to the subtype of leukemia and WBC levels, but may not be related to age. BMT may not improve survival in these patients. More cases should be accumulated and summarized to better understand the profile in *KMT2A-SEPT6*-positive AML.

## Data availability statement

The raw data supporting the conclusions of this article will be made available by the authors, without undue reservation.

## Ethics statement

The studies involving human participants were reviewed and approved by the Ethics Committee of Shengjing Hospital of China Medical University. Written informed consent to participate in this study was provided by the participants' legal guardian/next of kin.

## Author contributions

SF and FC performed the study concept and design. FC developed the methodology and wrote the paper. FC and YY acquired, analyzed and interpreted the data, and performed the statistical analysis. SF reviewed and revised the paper. All authors read and approved the final paper.

## Funding

This work was supported by the National Natural Science Foundation of China (NSFC) [grant number: 82070165] and 345 Talent Project of Shengjing Hospital [grant number: M0957].

## Conflict of interest

The authors declare that the research was conducted in the absence of any commercial or financial relationships that could be construed as a potential conflict of interest.

## Publisher's note

All claims expressed in this article are solely those of the authors and do not necessarily represent those of their affiliated organizations, or those of the publisher, the editors and the reviewers. Any product that may be evaluated in this article, or claim that may be made by its manufacturer, is not guaranteed or endorsed by the publisher.

## References

[1] Culp-Hill R D'Alessandro A Pietras EM. Extinguishing the embers: targeting AML metabolism. Trends Mol Med. (2021) 27:332–44. 10.1016/j.molmed.2020.10.001 33121874 PMC8005405

[2] Hasserjian RP. Controversies in the recent (2016) World Health Organization classification of acute myeloid leukemia. Best Pract Res Clin Haematol. (2021) 34:101249. 10.1016/j.beha.2021.101249 33762104

[3] Matsuo H Yoshida K Fukumura K Nakatani K Noguchi Y Takasaki S. Recurrent CCND3 mutations in MLL-rearranged acute myeloid leukemia. Blood Adv. (2018) 2:2879–89. 10.1182/bloodadvances.2018019398 30381403 PMC6234363

[4] Wander P Arentsen-Peters STCJM Pinhan?os SS Koopmans B Dolman MEM Ariese R. High-throughput drug screening reveals Pyrvinium pamoate as effective candidate against pediatric MLL-rearranged acute myeloid leukemia. Transl Oncol. (2021) 14:101048. 10.1016/j.tranon.2021.101048 33667892 PMC7933809

[5] Meyer C Burmeister T Gröger D Tsaur G Fechina L Renneville A. The MLL recombinome of acute leukemias in 2017. Leukemia. (2018) 32:273–84. 10.1038/leu.2017.213 28701730 PMC5808070

[6] Ono R Taki T Taketani T Kawaguchi H Taniwaki M Okamura T. SEPTIN6, a human homolog to mouse Septin6, is fused to MLL in infant acute myeloid leukemia with complex chromosomal abnormalities involving 11q23 and Xq24. Cancer Res. (2002) 62:333–7. 11809673

[7] Ivanov AI Le HT Naydenov NG Rieder F. Novel functions of the septin cytoskeleton: shaping up tissue inflammation and fibrosis. Am J Pathol. (2021) 191:40–51. 10.1016/j.ajpath.2020.09.007 33039354 PMC7786077

[8] Macara IG Baldarelli R Field CM Glotzer M Hayashi Y Hsu SC. Mammalian septins nomenclature. Mol Biol Cell. (2002) 13:4111–3. 10.1091/mbc.e02-07-0438 12475938 PMC138619

[9] Cerveira N Bizarro S Teixeira MR. MLL-SEPTIN gene fusions in hematological malignancies. Biol Chem. (2011) 392:713–24. 10.1515/BC.2011.072 21714766

[10] Wei Y Yang J Yi L Wang Y Dong Z Liu Z. MiR-223-3p targeting SEPT6 promotes the biological behavior of prostate cancer. Sci Rep. (2014) 4:7546. 10.1038/srep07546 25519054 PMC4269886

[11] Ono R Ihara M Nakajima H Ozaki K Kataoka-Fujiwara Y Taki T. Disruption of Sept6, a fusion partner gene of MLL, does not affect ontogeny, leukemogenesis induced by MLL-SEPT6, or phenotype induced by the loss of Sept4. Mol Cell Biol. (2005) 25:10965–78. 10.1128/MCB.25.24.10965-10978.2005 16314519 PMC1316963

[12] Borkhardt A Teigler-Schlegel A Fuchs U Keller C König M Harbott J. An ins(X;11)(q24;q23) fuses the MLL and the Septin 6/KIAA0128 gene in an infant with AML-M2. Genes Chromosomes Cancer. (2001) 32:82–8. 10.1002/gcc.1169 11477664

[13] Slater DJ Hilgenfeld E Rappaport EF Shah N Meek RG Williams WR. MLL-SEPTIN6 fusion recurs in novel translocation of chromosomes 3, X, and 11 in infant acute myelomonocytic leukemia and in t(X;11) in infant acute myeloid leukemia, and MLL genomic breakpoint in complex MLL-SEPTIN6 rearrangement is a DNA topoisomerase II cleavage site. Oncogene. (2002) 21:4706–14. 10.1038/sj.onc.1205572 12096348

[14] Kim HJ Ki CS Park Q Koo HH Yoo KH Kim EJ. MLL/SEPTIN6 chimeric transcript from inv ins(X;11)(q24;q23q13) in acute monocytic leukemia: report of a case and review of the literature. Genes Chromosomes Cancer. (2003) 38:8–12. 10.1002/gcc.10235 12874781

[15] Fu JF Liang DC Yang CP Hsu JJ Shih LY. Molecular analysis of t(X;11)(q24;q23) in an infant with AML-M4. Genes Chromosomes Cancer. (2003) 38:253–9. 10.1002/gcc.10272 14506700

[16] Harrison CJ Cuneo A Clark R Johansson B Lafage-Pochitaloff M Mugneret F. Ten novel 11q23 chromosomal partner sites. European 11q23 workshop participants. Leukemia. (1998) 12:811–22. 10.1038/sj.leu.2401017 9593286

[17] Nakata Y Mori T Yamazaki T Suzuki T Okazaki T Kurosawa Y. Acute myeloid leukemia with hypergranular cytoplasm accompanied by t(X;11)(q24;q23) and rearrangement of the MLL gene. Leuk Res. (1999) 23:85–8. 10.1016/S0145-2126(98)00131-3 9933140

[18] Cerveira N Lisboa S Correia C Bizarro S Santos J Torres L. Genetic and clinical characterization of 45 acute leukemia patients with MLL gene rearrangements from a single institution. Mol Oncol. (2012) 6:553–64. 10.1016/j.molonc.2012.06.004 22846743 PMC5528393

[19] De Braekeleer E Meyer C Douet-Guilbert N Basinko A Le Bris MJ Morel F. Identification of MLL partner genes in 27 patients with acute leukemia from a single cytogenetic laboratory. Mol Oncol. (2011) 5:555–63. 10.1016/j.molonc.2011.08.003 21900057 PMC5528326

[20] Cerveira N Micci F Santos J Pinheiro M Correia C Lisboa S. Molecular characterization of the MLL-SEPT6 fusion gene in acute myeloid leukemia: identification of novel fusion transcripts and cloning of genomic breakpoint junctions. Haematologica. (2008) 93:1076–80. 10.3324/haematol.12594 18492691

[21] Kadkol SS Bruno A Oh S Schmidt ML Lindgren V. MLL-SEPT6 fusion transcript with a novel sequence in an infant with acute myeloid leukemia. Cancer Genet Cytogenet. (2006) 68:162–7. 10.1016/j.cancergencyto.2006.02.020 16843108

[22] Arber DA Brunning RD Le Beau MM. Acute myeloid leukemia and related precursor neoplasms. In: Swerdlow SH, Campo E, Harris NL, et al. editors. WHO Classification of Tumors of Haematopoietic and Lymphoid Tissues. Revised 4th ed. France: IARC Press (2017). p. 130–71.

[23] Nguyen S Leblanc T Fenaux P Witz F Blaise D Pigneux A. A white blood cell index as the main prognostic factor in t(8;21) acute myeloid leukemia (AML): a survey of 161 cases from the French AML Intergroup. Blood. (2002) 99:3517–23. 10.1182/blood.V99.10.3517 11986202

[24] Antunes ETB Ottersbach K. The MLL/SET family and haematopoiesis. Biochim Biophys Acta Gene Regul Mech. (2020) 1863:194579. 10.1016/j.bbagrm.2020.194579 32389825 PMC7294230

[25] Rice S Roy A. MLL-rearranged infant leukemia: a 'thorn in the side' of a remarkable success story. Biochim Biophys Acta Gene Regul Mech. (2020) 1863:194564. 10.1016/j.bbagrm.2020.194564 32376390

[26] Balgobind BV Zwaan CM Pieters R Van den Heuvel-Eibrink MM. The heterogeneity of pediatric MLL-rearranged acute myeloid leukemia. Leukemia. (2011) 25:1239–48. 10.1038/leu.2011.90 21566656

[27] Sachs Z LaRue RS Nguyen HT Sachs K Noble KE Mohd Hassan NA. NRASG12V oncogene facilitates self-renewal in a murine model of acute myelogenous leukemia. Blood. (2014) 124:3274–83. 10.1182/blood-2013-08-521708 25316678 PMC4239336

[28] Trentin L Bresolin S Giarin E Bardini M Serafin V Accordi B. Deciphering KRAS and NRAS mutated clone dynamics in MLL-AF4 pediatric leukemia by ultra deep sequencing analysis. Sci Rep. (2016) 6:34449. 10.1038/srep34449 27698462 PMC5048141

[29] Bacher U Haferlach T Schoch C Kern W Schnittger S. Implications of NRAS mutations in AML: a study of 2502 patients. Blood. (2006) 107:3847–53. 10.1182/blood-2005-08-3522 16434492

[30] Wang S Wu Z Li T Li Y Wang W Hao Q. Mutational spectrum and prognosis in NRAS-mutated acute myeloid leukemia. Sci Rep. (2020) 10:12152. 10.1038/s41598-020-69194-6 32699322 PMC7376066

[31] Wong NM So CWE. Novel therapeutic strategies for MLL-rearranged leukemias. Biochim Biophys Acta Gene Regul Mech. (2020) 1863:194584. 10.1016/j.bbagrm.2020.194584 32534041

[32] Welch JS Ley TJ Link DC Miller CA Larson DE Koboldt DC. The origin and evolution of mutations in acute myeloid leukemia. Cell. (2012) 150:264–78. 10.1016/j.cell.2012.06.023 22817890 PMC3407563

[33] Liu X Ye Q Zhao XP Zhang PB Li S Li RQ. RAS mutations in acute myeloid leukemia patients: a review and meta-analysis. Clin Chim Acta. (2019) 489:254–60. 10.1016/j.cca.2018.08.040 30194935

[34] Damm F Heuser M Morgan M Wagner K Görlich K Grosshennig A. Integrative prognostic risk score in acute myeloid leukemia with normal karyotype. Blood. (2011) 117:4561–8. 10.1182/blood-2010-08-303479 21372155

[35] Li TT Li J Geng YH Zhang F Liu L Yang YL. NRAS gene expression and its clinical significance in patients with acute myeloid leukemia. Zhongguo Shi Yan Xue Ye Xue Za Zhi. (2020) 28:76–81. 10.19746/j.cnki.issn.1009-2137.2020.01.013 32027256

[36] Winters AC Bernt KM. MLL-rearranged leukemias-an update on science and clinical approaches. Front Pediatr. (2017) 5:4. 10.3389/fped.2017.00004 28232907 PMC5299633

[37] Balgobind BV Raimondi SC Harbott J Zimmermann M Alonzo TA Auvrignon A. Novel prognostic subgroups in childhood 11q23/MLL-rearranged acute myeloid leukemia: results of an international retrospective study. Blood. (2009) 114:2489–96. 10.1182/blood-2009-04-215152 19528532 PMC2927031

